# Sharing and Allocation in Preschool Children: The Roles of Theory of Mind, Anticipated Emotions, and Consequential Emotions

**DOI:** 10.3390/bs14100931

**Published:** 2024-10-11

**Authors:** Yingdi Shi, Mengnan Zhang, Liqi Zhu

**Affiliations:** 1 CAS Key Laboratory of Behavioral Science, Institute of Psychology, Chinese Academy of Sciences, Beijing 100101, China; feifeilujie@gmail.com (Y.S.); zhangmengnan@psych.ac.cn (M.Z.); 2 Department of Psychology, University of Chinese Academy of Sciences, Beijing 100101, China

**Keywords:** theory of mind, anticipated emotions, consequential emotions, sharing, resource allocation

## Abstract

This study investigates the impact of theory of mind, anticipated emotions before actual behavior, and consequential emotions following the behavior on sharing and allocation behavior in 4–6-year-old children. In Experiment 1, 95 children were randomly assigned to three conditions (external emotion expectancy condition, internal emotion expectancy condition, and control condition) to explore the role of cognition and emotions in children’s sharing and allocation behaviors. Experiment 2 employed a dictator game to further validate the influence of theory of mind and consequential emotions on behavior. The findings indicated that both anticipated and consequential emotions influence sharing behavior, but neither serves as a key predictor of allocation behavior. Theory of mind influences children’s sharing behavior and is related to the fairness of allocation. Children with higher levels of theory of mind tend to rate consequential emotions more positively, while those with lower ratings of consequential emotions are more likely to reconsider sharing after reflection. Notably, theory of mind and emotional factors demonstrate distinct motivational effects on children’s prosocial sharing and resource allocation, with negative emotions exhibiting a more pronounced impact on decision-making processes.

## 1. Introduction

Sharing and allocation behaviors play crucial roles in fostering social cooperation, establishing prosocial relationships, and shaping fairness and justice [1,2,3]. However, existing research often conflates these behaviors, such as using resource allocation experimental designs to study sharing behavior [4,5,6], or failing to distinctly differentiate between the two in drawing experimental conclusions [7,8], potentially leading to misjudgments regarding the developmental characteristics of children’s behaviors. Despite their similarities, children’s understanding of resource ownership differs between sharing and allocation [9]. Sharing involves individual resources, while allocation involves collective resources. The better children understand ownership, the more frequent and rapid their sharing behaviors [10], whereas in contexts where resource ownership is not explicitly defined, allocation behaviors exhibit consistency [4,11]. This study aims to distinguish resource ownership in these behaviors and explore their primary influencing factors.

Cognitive and affective factors play pivotal roles in decision making, mutually influencing and interacting [12,13,14]. This study will investigate children’s developmental changes in sharing and allocation decision making from cognitive and emotional perspectives.

### 1.1. The Influence of Theory of Mind on Sharing and Allocation Behaviors

Theory of Mind (ToM) refers to the attribution of mental states (such as beliefs, intentions, thoughts, and emotions) to oneself or others, and the capacity to comprehend and predict the behavior of others. It is considered a pivotal factor in the cognitive development of preschool children [15]. Although ToM has been associated with altruism and fair decision making, conflicting results exist in current research. Some studies have found a positive correlation between ToM and sharing behavior [16], while others have observed reduced sharing behavior in children with higher levels of ToM [17], or have found no significant correlation between the two [18]. Regarding allocation behaviors, ToM has been regarded as a predictive factor for fairness [19,20]; however, other research suggests that ToM is unrelated to fairness and only influences children’s decisions to accept equal distributions [1]. These contradictory findings further indicate the intricate relationship between ToM and sharing and allocation behaviors.

### 1.2. The Influence of Anticipated and Consequential Emotions on Sharing and Allocation Behaviors

Emotional factors play a pivotal role in decision-making processes related to sharing and allocation [2,21,22,23]. Anticipated emotions preceding the behavior [24,25] and consequential emotions following the behavior [26,27,28] both exert influence on prosocial behaviors [2,23]. Research indicates that children of varying age groups demonstrate differences in emotional reliance, with younger children exhibiting a greater reliance on consequential emotions, while older children display a predilection towards anticipated emotions [29]. Nevertheless, investigations into the specific mechanisms underlying these two types of emotions and their cultural variations remain relatively scarce, predominantly drawing from Western cultural samples [2,22]. This study aims to address this gap by, for the first time, examining the specific impact processes of anticipated and consequential emotions on behavioral decision making within an Eastern cultural context.

Previous research has often examined sharing and allocation behaviors from the perspective of resource sharers. For instance, Paulus and Moore (2015) emphasized the consideration of children as resource sharers regarding the potential emotional reactions of recipients when making allocation decisions [22]. Other studies have also found that anticipating negative emotions in response to unfair behavior increases the likelihood of fair decision making [30]. Conversely, consequential emotions also influence individual behavioral decisions [31], feelings from actual behavioral outcomes influence ethical choices and behaviors [25], and consequential emotions after rule violations predict allocation in dictator games [32], and children are more likely to choose fairness after observing disappointed emotional responses [27]. However, decision making is often related to others, and the roles of resource sharers and recipients differ [33]. Emotional perspectives for imagining others’ feelings differ from those for imagining one’s own feelings [34], and decisions about sharing and allocating behaviors may differ. Therefore, this study simultaneously examines the emotional influences of both sharers and recipients on children’s sharing and allocation behaviors, while incorporating a control group to ascertain whether children’s actual decisions are influenced by anticipated emotions from different perspectives (Experiment 1) and consequential emotions (Experiment 2).

### 1.3. The Relationship between ToM and Emotional Factors

Existing research has illuminated the connection between ToM and emotional understanding [14,35,36]. The advancement of ToM is closely associated with children’s accurate understanding of others’ intentions [37], and a high level of ToM capacity enables children to flexibly consider the intentions behind the behavior [38]. Furthermore, the emotion appraisal theory model emphasizes the pivotal role of emotional information in inferring others’ mental states [39]. Individuals retrieve relevant information during emotional expression to assess and infer others’ emotional states [40], while others’ emotional reactions can also influence individuals’ evaluations of events and emotional responses [30].

Based on this, we hypothesize that there is a dynamic interaction between ToM and emotional factors, which may impact children’s sharing and allocation behaviors. However, the specific relationship between the two, as well as their combined mechanisms in influencing behavioral decisions, requires further investigation. Previous studies have shown that ages 4 to 5 are critical for developing ToM in children [16,17]. By the age of 6, children’s level of ToM is already higher than the randomized level [41]. Therefore, ages 4 to 6 might represent an important stage for the development of ToM in children. Additionally, a meta-analysis has suggested that emotional factors and prosocial behavior are correlated from preschool to adolescence [42]. Previous research has also provided evidence for the relationship between emotional factors from the recipient’s perspective and behavior in children aged 4 to 6 [22,43]. Based on prior developmental studies on participant selection in sharing and allocation behaviors [17,23,44], it is of significant importance to explore how ToM and emotional factors influence the development of sharing and allocation in children aged 4 to 6. Therefore, this study selects preschool children aged 4 to 6 as the research subjects. It aims to explore, through experimental methods, how ToM and emotional factors (including anticipated emotions and consequential emotions) influence sharing and allocation decisions in children at this developmental stage.

### 1.4. Current Studies

This study aims to investigate the impact of ToM, anticipated emotions, and consequential emotions on sharing and allocation decisions among Chinese children aged 4–6 comparatively. Experiment 1 examines the influence of different emotional expectations on children’s sharing and allocation behaviors by distinguishing resource ownership, further delving into the relationship between ToM and anticipated emotions. Given previous research indicating that negative emotional expectations have a greater impact on children’s sharing and allocation decisions than positive emotions [2,22,23], this study focuses on the influence of anticipated emotions in non-sharing scenarios on behavioral decisions. The “external emotional expectation condition (hereinafter referred to as ‘E-Predict’)” primarily investigates whether children, as sharers, anticipate the emotions of the resource recipients when they choose not to share with others, and how this anticipation may influence their behavioral decisions. The “internal emotional expectation condition (hereinafter referred to as ‘I-Predict’)” investigates scenarios where children, as recipients, are not shared with. The “control condition (hereinafter referred to as ‘C-Control’)” examines children’s sharing or allocation behavior without emotional interference [45]. Our hypothesis is:

**H1a.**  *There are differences in the emotional anticipation among the three conditions of children. In the E-Predict and the I-Predict, children’s expectations of the recipient’s negative emotions increase their willingness to share and allocate resources. Compared to the C-Control, children in the E-Predict and I-Predict conditions are hypothesized to demonstrate greater generosity in sharing and allocation behaviors*.

**H1b.**  *The level of ToM positively predicts children’s decisions regarding sharing and allocation, and children with a high level of development of ToM are more inclined to fair sharing and allocation*.

**H1c.**  *ToM affects children’s emotional expectations of recipients. Children with high scores of ToM have more negative emotional expectations of recipients and then show more sharing and allocation behavior*.

Experiment 2 focuses on the impact of consequential emotions and ToM on sharing and allocation behavior, utilizing a voluntary sharing and allocation task. Following task completion, children rated the recipient’s emotions to examine the actual impact of consequential emotions on sharing and allocation decisions. We hypothesized the following:

**H2a.**  *Children’s ratings of the recipient’s consequential emotions are related to their behavioral decisions. Children's ratings of recipients' consequential emotions may predict their re-sharing and re-allocation*.

**H2b.**  *ToM influences children’s evaluation of recipients’ consequential emotions, and may also affect sharing and allocation behavior*.

## 2. Experiment 1: The Influence of Anticipated Emotions and ToM on Sharing and Allocation Behavior

### 2.1. Participants

Using G*Power 3.1 software to estimate the sample size, an effect size of 0.4, a significance level of 0.05, and a statistical power of 0.95 were determined, resulting in a required sample size of 72 participants. Further, 2 children did not complete the experiment due to problems with attention and the inability of the available stickers to meet preferences, and the final sample included 95 4 to 6-year-old preschool children (45 males, 50 females), with a mean age of 64.95 months (*SD* = 8.17). The sample included 28 four-year-old children (*M* = 55.5, *SD* = 2.7; 12 boys), 30 five-year-old children (*M* = 64.9, *SD* = 4.1; 16 boys), and 37 six-year-old children (*M* = 74.0, *SD* = 1.8; 18 boys). The participants were randomly assigned to the three conditions: the E-Predict condition (33 participants, M = 65.24 months, SD = 1.54), the I-Predict condition (31 participants, M = 65.61 months, SD = 1.53), and the C-Control condition (31 participants, M = 64.41 months, SD = 1.3). None of the children had previously participated in similar experiments, and they all obtained informed consent from their guardians and verbal consent from the children. Data collection took place from December 2023 to January 2024.

### 2.2. Procedures

The testing was conducted by a female experimenter in a quiet, independent room at the kindergarten, with the entire session being audio and video recorded. Standardized children’s images were used, with gender-matched pictures randomly selected. Children first learned to use a facial emotion scale and then completed two ToM tasks, namely the Unexpected Location Task and the Unexpected Content Task. Subsequently, the children were randomly assigned to participate in the emotional anticipation experiment, with each child participating in only one group. Finally, sharing and allocation tasks were administered, and the order of the two tasks was balanced. The total duration of the experiment was approximately 20 min.

### 2.3. Measures

#### 2.3.1. Emotion Judgment Task

**Figure 1:**
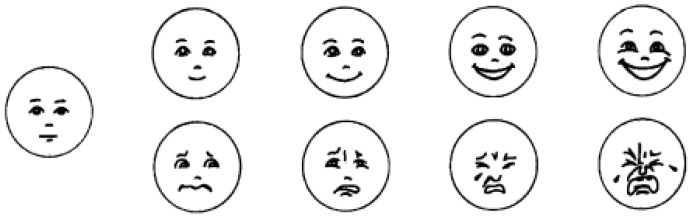
Facial Affect Scale.

We utilized the Facial Affect Scale [46] to assess children’s emotion ratings. This scale comprises nine consecutive facial expression illustrations, ranging from significantly sad to progressively happy, and the score is −4 to 4, with 0 representing a neutral emotional state. Experimental illustrations are depicted in Figure 1. The experimenter showed the children how to rate their emotions using the Facial Emotions Scale and asked the children to demonstrate how to use the scale. (For example, “How do you feel inside when your mom takes you out to play?/What do you feel inside when your toys get broken?”).

#### 2.3.2. ToM Task

ToM tasks were adapted from two classic false belief understanding tasks. The Unexpected Location Task was adapted from Baron-Cohen’s unexpected transfer of false belief task [37], using two photographs of children of the same gender and actual props to create the scenario: “Bobo saw Cindy put the candy in the box, then Cindy went out to play. Bobo took the candy out and put it in the bag. After a while, Cindy came back.” The experimenter posed three questions: “Where did Cindy initially put the candy? Where is the candy now? Where will Cindy look for the candy when she comes back?” If the children answered all questions correctly, they scored 1 point in the Unexpected Location Task; otherwise, they scored 0 points.

The Unexpected Content Task was adapted from the classic “Smarties” task [47]. In this task, the children saw a toothpaste box containing a marker inside. The experimenter also asked three questions: “What's in the box? What's inside the box when you open it? If another child saw the unopened box, what would he or she think was inside?” If the child could pass the consistency check of this task, they were considered to have passed the test and scored 1 point; otherwise, they scored 0 points.

Children who passed both tasks were classified as the ToM pass group, while those who did not pass were classified as the non-pass group.

#### 2.3.3. Anticipated Emotion Tasks

This study modified the anticipated emotion task based on previous research [22]. Children were grouped randomly by age. In the E-Predict condition, a photo of a boy or a girl was randomly chosen as the gift recipient. Initially, children were asked to assess the recipient’s emotions. Subsequently, they were presented with a scenario: “This child is called Max (Emma). He/she comes from another kindergarten. Imagine, one day he/she comes to your kindergarten to participate in a sharing activity. You have two candies (place the candies next to the participating child), but you do not want to share them with him/her. How do you think he/she feels?” Participants were then asked to rate Max’s (Emma’s) emotion using the Facial Emotion Scale. The difference between two emotion ratings is Anticipated Emotional Rating Difference Score (AERDS). In ‘E-Predict condition is ‘E-Predict condition’s AERDS.

In the I-Predict condition, a photo of a boy or a girl was randomly selected as the gift sharer. First, the child was invited to rate their own emotions as the recipient. Children were then presented with the following story: “This child is called Jack (Olivia). One day, Jack (Olivia) comes to the kindergarten to participate in a sharing activity. He/she brings two candies to you (place the candies next to the child). However, he/she refuses to share with you. How do you think you would feel?” The child was then invited to rate their own emotions as the recipient once again. The difference between the two emotion ratings represents the AERDS for the I-Predict condition.

In the C-Control condition, in line with previous research [45,48], children exclusively engaged in simple cognitive judgment tasks devoid of emotional intervention, for example, the child is shown a ball hidden inside a box and is then asked whether they know what it is inside. Before and after the cognitive judgment task, children were asked to rate their emotions using the Facial Affect Scale. Participants with two identical ratings were considered valid, and the difference between the two ratings was used to calculate the AERDS for the C-Control condition.

#### 2.3.4. Sharing Task

The sharing task was based on the dictator game paradigm [49], utilizing stickers as the primary resource for children’s sharing behavior [5,23]. During the experiment, children chose their favorite 6 out of 12 stickers to increase the variety of choices and mitigate the impact of preference on the results. Upon choosing the stickers, they were informed that the recipients were same-aged children from another kindergarten who also liked stickers and were shown a photo of the recipient.

Before sharing, children were explicitly informed that the resources belonged to them and that they had the autonomy to decide the extent of sharing. Once it was ensured that children understood the ownership and independent decision making regarding sharing, researchers refrained from observing to safeguard the privacy of the sharing process and minimize external influences on the children’s choices. Subsequently, children placed the selected stickers into two distinct colored envelopes, symbolizing sharing and personal retention. If children did not understand resource ownership or the game rules during the experiment, the experimenter would explain them again to ensure children’s understanding.

We recorded the number of stickers the child shared with the recipient and credited 1 point per sticker. Based on the fairness of the sharing, children who shared 3 stickers were recorded as a fair group and otherwise as a non-fair group.

#### 2.3.5. Resource Allocation Task

The allocation task retained the framework of the sharing task but introduced the concept of joint ownership. Children chose their favorite 6 out of 12 stickers while being explicitly informed that these stickers were a joint gift from the teacher to them and another child and were invited to make an allocation. Once the children confirmed their understanding of the shared ownership, the experimenter turned away, and the children proceeded with the allocation task. If children answered incorrectly about ownership, the experimenter would explain it again to ensure their understanding, and then turn around to invite the children to complete the allocation task.

We recorded the number of stickers the child allocated to the recipient and credited 1 point per sticker. Based on the fairness of the allocation, children who allocated 3 stickers were classified as the fair group, while all other instances were categorized as the non-fair group.

#### 2.3.6. Data Analysis

We utilized SPSS 26.0 for the analysis. Initially, we employed the Shapiro–Wilk test for exploratory analysis. The quantity of sharing and allocation in the three emotional anticipation groups did not conform to a normal distribution (*p* < 0.05). Therefore, we opted for non-parametric testing methods for subsequent analysis. When analyzing the relationship between ToM, anticipated emotions, and the amount of sharing in the E-Predict condition, we chose hierarchical linear regression for analysis, as the residuals met the assumption of normal distribution.

### 2.4. Results

#### 2.4.1. Analyzing the Differential Impact of Anticipated Emotions on Sharing and Allocation Behavior

**Figure 2:**
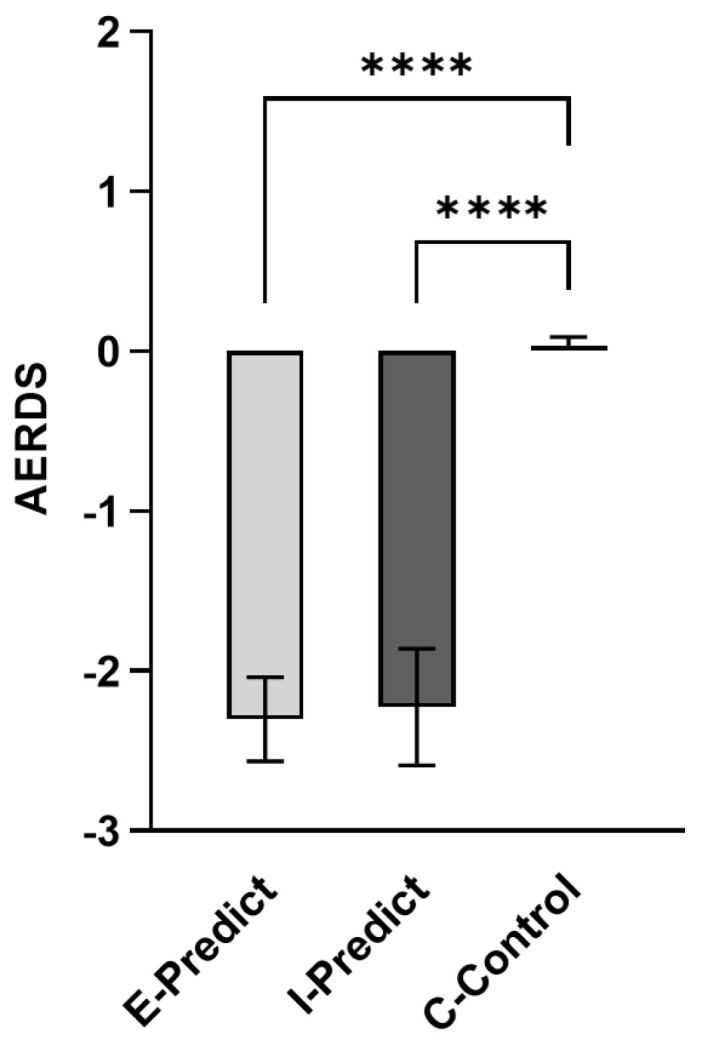
Comparison of Anticipation Emotional Rating Difference Score among three conditions of children, **** *p* < 0.0001.

We first examined children’s understanding of recipients’ emotions; gender had no significant effect on children’s AERDS, Mann–Whitney *Z* = −0.62, *p* = 0.536, the Kruskal–Wallis rank-sum test found that age had no significant effect on children’s AERDS (E-Predict *H* = 6.57, *p* = 0.751; I-Predict *H* = 4.56, *p* = 0.103; C-Control *H* = 0.22, *p* = 0.896), confirming the effectiveness of random grouping. Furthermore, Kruskal–Wallis H tests unveiled a significant difference in AERDS among the three conditions of children (*H* = 50.31, *p* < 0.001). Bonferroni-corrected multiple comparisons revealed significant differences between the E-Predict condition and the C-Control condition (*p* < 0.001), as well as between the I-Predict condition and the C-Control condition (*p* < 0.001), with no significant difference observed between the two anticipated emotion conditions (*p* = 0.730 > 0.05) (see Figure 2).

In the sharing task, the Kruskal–Wallis rank-sum test revealed a significant difference in the sharing quantity among children in the three emotion anticipation conditions (*H* = 7.08, *p* = 0.029 < 0.05). Compared to the C-Control condition, children in both the E-Predict condition and the I-Predict condition showed a greater tendency to share. post hoc Bonferroni multiple comparisons indicated a significant difference in sharing quantity between the I-Predict condition and the C-Control condition (*p* = 0.043 < 0.05).

In the allocation task, significant differences in allocation quantity were observed among children in the three emotion anticipation conditions (*H* = 6.48, *p* = 0.039 < 0.05). Children in the E-Predict condition and the I-Predict condition were allocated more than children in the C-Control condition. Furthermore, a significant difference in allocation quantity was found between children in the I-Predict condition and the C-Control condition (*p* = 0.033 < 0.05) (see Table 1).

#### 2.4.2. The Role of ToM

The results showed that mastery of ToM increased with children’s age. Although the overall difference did not reach statistical significance (*χ*^2^ (2, *N* = 95) = 5.45, *p* = 0.065), performance on the Unexpected Location Task was significantly correlated with age (*χ*^2^ (2, *N* = 95) = 11.68, *p* = 0.003), implying that the development of ToM is associated with specific cognitive abilities Related.

A Mann–Whitney rank sum test revealed that the level of ToM has a significant impact on children’s sharing behavior (*Z* = 1.98, *p* = 0.047 < 0.05), and children with high levels of ToM ability share significantly more than children with low levels of ToM ability. In the allocation task, there was no significant association between the level of ToM and the allocation amount (*Z* = −0.93, *p* = 0.354), as shown in Table 2. Further analyses found no statistically significant differences between the Unexpected Content Task and the Unexpected Location Task, nor the amount of children’s sharing or the amount of allocation.

In addition, the chi-square test revealed a marginal trend between the level of children’s ToM and the fairness of sharing, *χ*^2^ (1, *N* = 95) = 3.68, *p* = 0.055, and children with a higher level of ToM were more significant in allocation justice, *χ*^2^ (1, *N* = 95) = 4.96, *p* = 0.026.

#### 2.4.3. The Relationship between ToM, Anticipated Emotions, and Sharing Behavior

Using Spearman correlation analysis, the study explored the correlation between children’s anticipated emotions, ToM, and sharing behavior. In the E-Predict condition, ToM did not impact the children’s AERDS (*r* = −0.272, *p* = 0.126 > 0.05). The negative correlation between AERDS and sharing behavior (*r* = −0.360, *p* = 0.039 < 0.05) suggested that in situations where children did not share, their anticipation of negative emotions in the recipient was significantly associated with a higher sharing quantity, hinting at the possibility that negative anticipation may have facilitated sharing behavior. In the I-Predict condition, ToM did not affect the children’s AERDS (*r* = −0.113, *p* = 0.545 > 0.05), and AERDS did not impact sharing behavior (*r* = −0.144, *p* = 0.441 > 0.05). Similarly, in the C-Control condition, ToM did not influence AERDS (*r* = −0.070, *p* = 0.709 > 0.05), and the relationship between AERDS and sharing behavior was not significant (*r* = 0.332, *p* = 0.068 > 0.05).

The hierarchical linear regression analysis in Table 3 further confirmed a negative impact of AERDS (*β* = −0.388, *p* = 0.026 < 0.05) on sharing behavior in the E-Predict condition after controlling for age. This finding suggested that young children were more inclined to engage in sharing when anticipating negative emotions from not sharing. Although no significant predictive relationship was found between levels of ToM and sharing behavior (*β* = 0.156, *p* = 0.360), the maturity of ToM and the ability to understand others’ emotional states were closely linked, potentially indirectly influencing sharing decisions.

#### 2.4.4. The Relationship between ToM, Anticipated Emotions, and Allocation Behavior

We used spearman correlation analysis to examine the relationships between anticipated emotion, ToM, and allocation behavior in children. In the E-Predict condition, ToM did not affect children’s anticipated emotion difference (*r* = −0.272, *p* = 0.126 > 0.05). There was a marginal correlation between anticipated emotion difference and allocation behavior (*r* = −0.343, *p* = 0.050). In the I-Predict condition, ToM did not influence children’s anticipated emotion difference (*r* = −0.113, *p* = 0.545 > 0.05), and anticipated emotion difference also did not affect allocation behavior (*r* = 0.142, *p* = 0.447 > 0.05). In the C-Control condition, ToM did not impact anticipated emotion difference (*r* = −0.070, *p* = 0.709 > 0.05), and the relationship between anticipated emotion difference and allocation behavior was not significant (*r* = 0.041, *p* = 0.828 > 0.05).

Since there was a marginal correlation between anticipated emotion difference and allocation behavior in the E-Predict condition only, we conducted further analysis in this condition. The results showed that children’s ToM significantly predicted allocation fairness (*χ*^2^ (1, *N* = 33) = 8.19, *p* = 0.04), indicating a greater tendency for fair allocation among children with higher levels of ToM. Furthermore, logistic regression analysis controlling for age revealed a significant positive impact of ToM on fairness in allocation (*OR* = 11.54, 95% CI [1.73, 77.08], *p* = 0.012), while the effects of AERDS on fairness in allocation were not significant (*OR* = 0.96, 95% CI [0.50, 1.83], *p* = 0.897), as shown in Table 4.

### 2.5. Discussion

Experiment 1 investigates the impact of ToM and anticipated emotions on preschool children’s sharing and allocation decisions. Previous research has demonstrated that preschool children can anticipate emotions and base their social behavior decisions on this ability, supporting earlier findings on children’s emotional anticipation skills [22,23]. The anticipated emotional responses of the three conditions of children differ. In non-sharing scenarios, children in the E-Predict and I-Predict conditions exhibited higher levels of sharing and allocation compared to the C-Control condition, indicating that negative emotional anticipations may stimulate sharing and allocation behavior in young children. These behavioral variances may be linked to children’s comprehension of others’ emotions and self-attention levels. When not sharing, the emotional response to the recipient influences their subsequent behavioral decisions. The findings supported hypothesis H1a. Previous studies have established a connection between children’s emotional assessment and subsequent sharing behavior [23]. Our research expands upon and validates this conclusion. For the first time, it has been discovered that the ability to consider others’ emotions also impacts children’s generosity when not sharing with others.

This study revealed that children with high levels of ToM exhibit a greater propensity to share compared to those with lower levels of ToM, alongside displaying heightened fairness in allocation tasks. These findings indicate that the ToM capacity not only fosters children’s sensitivity to the needs of others but also bolsters their inclination to adhere to principles of fairness, thereby supporting hypothesis H1b. ToM is an evolving capacity, while many previous studies have posited it as a psychological mechanism influencing sharing behavior [5,6,16,50,51]. This study validated the finding, and also found that the correlation between the level of development of specific ToM tasks (e.g., the Unexpected Location Task or the Unexpected Content task) and sharing behavior was insignificant. This result implies that different components of the ToM may have different effects on children’s specific social behaviors, with some aspects of the ToM (e.g., empathy for others’ affective states) more directly contributing to sharing behaviors. In contrast, other elements (e.g., understanding of knowledge states) may not directly relate to sharing motivation. This suggests that the plurality of ToM and the specificity of assessment tasks must be more carefully considered in future research to better understand children’s social behavior and moral–emotional development. In addition, unlike sharing behavior, ToM did not affect children’s allocation willingness and quantity, which may be due to the weakened influence of anticipated emotions on young children’s allocation behavior when ownership of resources is shared. However, higher fairness was shown in allocation tasks, suggesting that ToM competence is an essential psychological mechanism influencing children’s child fairness development [20].

Hypothesis H1c points out that ToM affects anticipated emotions towards recipients, but the experimental results do not find a significant correlation between ToM and anticipated emotions. However, in the E-Predict condition, there is a specific relationship between the level of children’s ToM and their negative expectations of the recipient’s emotions, which may indicate that ToM affects the formation of emotional expectations in specific situations.

## 3. Experiment 2: The Effect of Consequential Emotion and ToM on Sharing and Allocation Behavior

### 3.1. Participants

The final sample included 61 4-to-6-year-old children (26 males and 35 females) with a mean age of 66.59 months (SD = 8.30). The sample included 17 four-year-old children (*M* = 55.1, *SD* = 2.6; 8 boys), 12 five-year-old children (*M* = 64.8, *SD* = 4.3; 4 boys), and 32 six-year-old children (*M* = 73.4, *SD* = 1.1; 14 boys). The sample selection criteria were the same as in Experiment 1, and the data were collected from December 2023 to January 2024.

### 3.2. Procedures

Experiment 2 followed the design framework of Experiment 1, with the critical difference being the replacement of the anticipated emotion task with the consequential emotion task, placed after the sharing and allocation task.

### 3.3. Measures

The emotion judgment task, the ToM task, and the share and allocation tasks were the same as in Experiment 1. In Experiment 2, children initially rated the standardized recipient avatar’s emotions. After sharing or allocating, they participated in a consequential emotion task, re-rated the recipient’s children’s emotions, and asked them about the possible response to the recipient’s emotions. For instance, following the sharing task, children were asked if they would share again based on their understanding of the recipient’s emotions. If children agreed to share again, the amount the child shared again was recorded as the re-sharing score. The initial sharing score was subtracted from the re-sharing score to calculate the Re-Sharing Score Difference (RSSD), which measures the extent to which participants differed in the two behaviors. The difference between the two emotional ratings was defined as the Consequential Emotion Rating Difference Score (CERDS). Allocation behavior is recorded similarly, with the allocation score difference being the Re-Allocation Score Difference (RASD).

Throughout the process, experimenters maintained a neutral stance toward the children’s behavior and refrained from intervening. While structurally similar to the sharing task, the allocation task clearly delineated ownership of resources and instructional language.

### 3.4. Results

#### 3.4.1. An Analysis of Consequential Emotions and Their Differential Impact on Sharing and Allocation Behaviors

**Figure 3:**
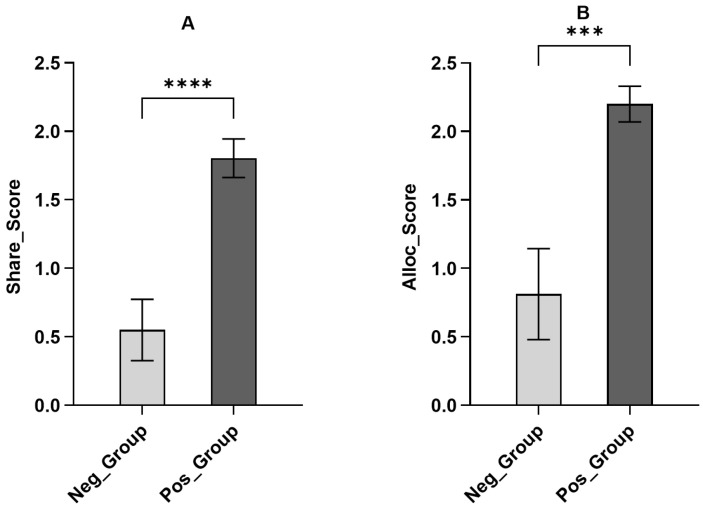
Comparison of the amount of sharing and allocation between children in the negative and positive groups. Error bars represent standard errors of the means. (**A**) Comparison of the amount of sharing between children in the negative and positive groups. (**B**) Comparison of the amount of allocation between children in the negative and positive groups. (*** *p* < 0.001, **** *p* < 0.0001).

This study categorized children into positive and negative groups based on their consequential emotional ratings toward the recipient after sharing. The aim was to explore the influence of emotional reactions on sharing and allocation behaviors. The Mann–Whitney rank sum test revealed significant differences in emotional ratings between the two groups of children for sharing behavior (*Z* = −6.40, *p* < 0.001) and allocation behavior (*Z* = −6.02, *p* < 0.001). Additionally, further Mann–Whitney rank sum tests explored significant differences in sharing behavior between the positive and negative groups (*Z* = −5.69, *p* < 0.001), and children in the positive and negative groups also differed significantly in RSSD (*Z* = −2.94, *p* = 0.003 < 0.05), with the negative group exhibiting significantly lower levels of sharing. In the allocation task, RASD between the positive and negative groups of children was not significant (*Z* = −1.05, *p* = 0.294 > 0.05). Children with negative consequential emotional ratings also allocated considerably fewer resources compared to children with positive emotional ratings (*Z* = −3.62, *p* < 0.001) (see Figure 3). These results indicate the significant role of consequential emotions in both the sharing and allocation behaviors.

#### 3.4.2. The Role of ToM

This study examined the mutual influence between ToM and CERDS through Mann–Whitney rank sum tests. In the sharing task, children exhibiting higher levels of ToM (*M* = 2.50, *Q*1 = 1.00, *Q*3 = 3.00) demonstrated more positive consequential emotional ratings compared to those with lower levels of ToM (*M* = 2.00, *Q*1 = −0.50, *Q*3 = 3.00), *Z* = −1.98, *p* = 0.048 < 0.05. However, in the allocation task there was no significant difference between ToM levels and consequential emotion ratings (*Z* = −0.20, *p* = 0.841 > 0.05), nor between ToM levels and CERDS (*Z* = −0.44, *p* = 0.658 > 0.05). Further exploring the potential influence of ToM levels on sharing and allocation behaviors following participation in consequential emotional tasks, the study found no significant associations between ToM levels and RSSD (*Z* = −1.77, *p* = 0.077 > 0.05) as well as RASD (*Z* = −0.33, *p* = 0.741 > 0.05). This suggests that the influence of ToM may depend on specific contexts and task types, and its effects may vary across different social behaviors.

#### 3.4.3. The Relationship between ToM, Consequential Emotions, and RSSD

The Spearman correlation analysis (see Table 5) showed a significant link between ToM and children’s CERDS responses in recipients (*r* = 0.256, *p* = 0.046 < 0.05). Additionally, recipients’ CERDS responses were significantly associated with children’s RSSD (*r* = −0.327, *p* = 0.01 < 0.05). This indicates that children with higher ToM levels are more likely to evoke positive emotional responses in recipients. Since there is no sharing differential for active group children in the sharing task, this notes that children who rated the recipient's consequential emotions positively did not re-share, children with negative ratings are more likely to change their behavioral decisions on reflection. For the children in the negative group, the Unexpected Location Task of ToM is significantly related to children’s willingness to share again (*r* = −0.510, *p* = 0.026 < 0.05). This suggests that those who performed well on the Unexpected Location Task of ToM showed a lower willingness to share after reflecting on the consequential emotions.

In further stratified linear regression analyses (see Table 6), after controlling for age, level of ToM and CERDS became key predictors of sharing behavior. At this point, neither ToM nor consequential emotions in the model showed a predictive effect on the sharing difference. Considering that children’s consequential emotions correlate with the CERDS, we conducted a correlation and regression analysis on this variable. We found that when the model used children’s consequential emotion ratings, a negative inward effect of consequential emotion ratings on RSSD (*β* = −0.325, *p* = 0.01 < 0.05) was found, emphasizing the direct association between emotion ratings and RSSD, with young children in the negative group being more likely to change their initial decision because of their perception of the recipient’s consequential emotion compared to young children in the positive group. However, the theory of mind itself was not shown to be a direct predictor of RSSD (*β* = −0.097, *p* = 0.439 > 0.05).

#### 3.4.4. The Relationship between ToM, Consequential Emotions, and RASD

Using Spearman’s correlation analysis, we explored the relationship between preschool children’s CERDS in recipients and their allocation behavior (see Table 7). The difference in children’s ratings of recipients’ consequential emotions (*r* = 0.09, *p* = 0.49 > 0.05) was not related to RASD. Additionally, there was no significant relationship between ToM and the CERDS (*r* = −0.027, *p* = 0.837 > 0.05) or RASD (*r* = −0.043, *p* = 0.744 > 0.05). As no significant correlations were found between the variables, further regression analysis was not pursued.

### 3.5. Discussion

Experiment 2 investigated the influence of ToM and consequential emotions on preschoolers’ sharing and allocation decisions. The results revealed significant differences in the behavior of children rated negatively or positively. Consequential emotions do not play a key role in allocative behavior. Following generous behavior, children could perceive the positive emotions of recipients, confirming the positive emotional benefits of generosity [23,52]. Moreover, after displaying positive behavior, children expressed higher consequential emotions satisfaction levels [53], and children also did not re-share or re-allocate. Simultaneously, we observed that following selfish behavior, children similarly perceived the negative emotional consequences of such behavior [22], and their understanding of these negative emotions influenced their behavioral tendencies afterwards. Expressing emotions triggers emotional responses and inferences from others and affects social interactions [31]. Judgments of consequential emotions are crucial factors influencing behavior [54], and understanding others' negative emotions through perspective-taking promotes prosocial behavior [55], partially supports supporting H2a.

Another research focus is the level of ToM and its influence on children’s expression of consequential emotions. In the sharing experiment involving sole ownership, children with higher ToM levels tended to rate consequential emotions more positively. On the contrary, children with lower levels of ToM rated the recipient’s consequential emotions more randomly. Children who gave lower ratings to the recipient’s emotions in the consequential emotion task tended to increase their prosocial behavior after reflecting on the consequential emotions. However, no relationship was found between the level of ToM and children’s willingness to share in subsequent tasks. In the allocation experiment involving shared ownership, for children with negative consequential emotion ratings, ToM was no longer a key factor influencing consequential emotions. At this point, CERDS also no longer predicted allocation behavior, which partially supports H2b. ToM only affects consequential emotions in specific situations. After the change in ownership, children’s perception of consequential emotions also underwent significant changes [56].

## 4. General Discussion

This study compared the influence of ToM, anticipated emotions, and consequential emotions on children’s sharing and allocation decisions. Early research predominantly focused on exploring the singular impact of cognitive factors [5,57,58] or emotional factors [22,59,60] on sharing or allocation behaviors. This study extended these early explorations by examining developmental patterns and psychological mechanisms within sharing and allocation behaviors [2,5,10,23].

While anticipated and consequential emotions operate at different junctures of judgment, they both negatively impact children’s sharing decisions when not sharing with the recipient. These findings complement prior research [2,22,60]. They support current theoretical perspectives, such as the emotion-guiding-behavior perspective [24,31] and the importance of emotional consequences for behavior control [54]. Anticipated negative emotions are more likely to evoke people’s memories and alter their behavioral decisions [61,62]. Previous behaviors provide cues for emotion rating and may also trigger emotional changes [63]. Behavior affects emotion [64], and emotion affects behavior again [31]. Negative emotions and behaviors play pivotal roles within this system.

In examining the influence of anticipated and consequential emotions on behavioral decisions, we found that in Experiment 1, children who imagined not sharing and anticipated more negative emotions from the recipient were more likely to share afterward. In Experiment 2, children who gave more positive ratings in the consequential emotion task were more willing to share in the free sharing task, while those with more negative ratings were more likely to change their sharing decisions after reflection. Christner et al. (2020) [2] previously hypothesized that anticipated and consequential emotions might have a similar influence on behavior, and our findings support this possibility. Children seem to realize that not sharing causes negative feelings for the recipient, and after considering this perspective, they become more inclined to share.

By exploring the relationship between ToM and emotional factors, this study initially revealed the relationship between ToM and two different emotions. In Experiment 1, children with high levels of ToM were more pessimistic about the recipient’s emotional anticipations when not sharing and tended to favor fairness in allocation behaviors. In Experiment 2, children with higher levels of ToM had more positive consequential emotions. The change in consequential emotions only affected sharing decisions and no longer influenced allocation decisions. Furthermore, acts of generosity and selfishness initially led to behavioral differentiation, revealing that not all preschoolers engaging in ToM tasks would generously share. After negative behavior, children who performed well in some branches of the ToM task would be more selfish, as observed in Cowell et al.’s study [17]. The nature of preschoolers’ behavioral differences may also be influenced by other factors, such as the development of individual cognition and language abilities [65], as well as children’s understanding of moral emotions [60], all of which are worthy of further exploration in future research.

The results of this study explored influences on sharing and allocating behaviors in terms of cognitive and emotional factors. They contribute to the developmental theory of sharing and allocation behavior. However, there are still some limitations that warrant attention. Children’s understanding of ToM and emotional factors is complex and adaptable throughout childhood, and the impact on behavior may vary across different stages, necessitating further longitudinal research to expand our conclusions. These also help us to sort out the potentially complex interactions between predictors more clearly. In Experiment 2, the autonomous sharing and allocation behavior naturally differentiated children into positive and negative groups, yet the factors leading to this difference require further exploration. Future studies could also induce positive or negative consequential emotions in children through experimental design to assess their impact on children’s behavioral decisions.

## 5. Conclusions

ToM and emotional factors (anticipated and consequential emotions) play a significant role in children’s sharing and allocation decisions. Children’s anticipation of negative emotions and emotional reflection on the results of negative behaviors as sharers will promote their prosocial sharing. Consequential emotions do not influence allocation behavior. ToM only affects allocation justice and the performance of children’s consequential emotional rating, thereby influencing sharing behavior. The study provides new insights into the diverse influencing factors that need to be addressed in future research. Childhood is a critical period for the development of prosocial and fair behaviors. Given that children’s behavioral decisions may benefit from their judgments and reflections on the recipient’s emotions, designing social skill training programs for preschool children and evaluating the impact of long-term interventions on their behavioral development holds significant research value.

## Figures and Tables

**Table 1:** Kruskal–Wallis rank-sum test for the amount shared and allocated by different conditions of children.

Variables	Conditions	Sample Size	M (P25, P75)	Kruskal–Wallis Rank-Sum Test	
				H	*p*
Amount of sharing	E-Predict	33	2.00 (1.00, 3.00)		
	I-Predict	31	2.00 (1.00, 3.00) *	7.08	0.029 *
	C-Control	31	1.00 (0.00, 2.00)		
Amount of allocation	E-Predict	33	2.00 (1.00, 3.00)		
	I-Predict	31	3.00 (1.50, 3.00) *	6.48	0.039 *
	C-Control	31	2.00 (0.50, 3.00)		

Note: * *p* < 0.05.

**Table 2:** Mann–Whitney rank-sum test for the amount of sharing and allocation of young children in different ToM conditions.

Variables	Conditions	Sample Size	M (P25, P75)	Mann–Whitney Rank-Sum Test	
				Z	*p*
Amount of sharing	0	62	1.00 (0.00, 2.00)	Z = −1.98	0.047 *
	1	33	2.00 (1.00, 3.00)		
Amount of allocation	0	62	2.00 (1.00, 3.00)	Z = −0.93	0.354
	1	33	3.00 (1.00, 3.00)		

Note: 0: group that did not pass the ToM, 1: group that passed the ToM, * *p* < 0.05.

**Table 3:** Linear regression of the E-Predict’ condition.

Predictive Variables	Sharing Behavior			
	Mode1		Mode2	
	β	*p*	β	*p*
age	0.256	0.150	0.255	0.126
ToM			0.156	0.360
AERDS			−0.388	0.026 *
R^2^ ΔR^2^ F	0.066 0.036 2.183	0.150 0.150	0.273 0.198 3.631	0.026 * 0.024 *

Note: * *p* < 0.05.

**Table 4:** Logistic regression for the E-Predict condition.

Variables	*β*	S.E.	*Z*	*p*	OR (95% CI)
Intercept	−1.43	1.18	−1.21	0.225	0.24 (0.02~2.41)
AERDS	−0.04	0.33	−0.13	0.897	0.96 (0.50~1.83)
Age					
4					1.00 (Reference)
5	0.99	1.16	0.85	0.393	2.68 (0.28~25.82)
6	−0.51	1.10	−0.47	0.641	0.60 (0.07~5.17)
ToM					
0					1.00 (Reference)
1	2.45	0.97	2.52	0.012 *	11.54 (1.73~77.08)

Note: OR: odds ratio, CI: confidence interval, * *p* < 0.05.

**Table 5:** Correlation analysis between young children’s consequential emotional ratings of recipients and sharing difference.

	1	2	3	4	5	6	7
1	1.000						
2	−0.006	1.000					
3	−0.120	0.404 **	1.000				
4	0.062	−0.038	0.403 **	1.000			
5	−0.005	0.251	0.694 **	0.794 **	1.000		
6	−0.057	0.227	0.224	0.120	0.256 *	1.000	
7	0.171	−0.218	−0.329 **	0.159	−0.228	−0.327 *	1.000

Note: 1: gender (0: female, 1: male); 2: age (4,5,6); 3: Unexpected Location Task (0: failed group, 1: passed group); 4: Unexpected Content Task (0: failed group, 1: passed group); 5: TOM (0: failed group, 1: passed group); 6: CERDS; 7: RSSD; (* *p* < 0.05, ** *p* < 0.01).

**Table 6:** Linear regression of ToM, Consequential emotions, and RSSD.

Predictive Variables	RSSD			
	Mode1		Mode2	
	β	*p*	β	*p*
age	−0.265	0.039	−0.178	0.176
ToM			−0.093	0.481
CERDS			−0.232	0.083
R^2^ ΔR^2^ F	0.070 0.054 4.456	0.039 0.039	0.138 0.092 3.034	0.117 0.036 *

Note: * *p* < 0.05.

**Table 7:** Correlation Analysis between Young Children’s Consequential Emotion Ratings of Recipients and RASD.

	1	2	3	4	5	6	7
1	1.000						
2	−0.006	1.000					
3	−0.120	0.404 **	1.000				
4	0.062	−0.038	0.403 **	1.000			
5	−0.005	0.251	0.694 **	0.794 **	1.000		
6	−0.006	0.036	0.014	0.033	0.058	1.000	
7	−0.043	−0.140	0.028	−0.095	−0.043	0.124	1.000

Note: 1: gender (0: female, 1: male); 2: age (4,5,6); 3: Unexpected Location Task (0: failed group, 1: passed group); 4: Unexpected Content Task (0: failed group, 1: passed group); 5: TOM (0: failed group, 1: passed group); 6: CERDS; 7: RASD; ** *p* < 0.01.

## Data Availability

The raw data supporting the conclusions of this article will be made available by the authors on request.
